# Protocol for Patch-Seq of Small Interneurons

**DOI:** 10.1016/j.xpro.2020.100146

**Published:** 2020-10-22

**Authors:** Marcela Lipovsek, Lorcan Browne, Matthew S. Grubb

**Affiliations:** 1 Centre for Developmental Neurobiology, Institute of Psychiatry, Psychology and Neuroscience (IoPPN), King’s College London, London, UK

## Abstract

Obtaining electrophysiological recordings and gene expression information from the same neuron (Patch-seq) brings forward a unique opportunity to study the transcriptional correlates of functional properties and vice versa. Here, we provide a detailed Patch-seq protocol tailored to the specialized demands of studying small interneurons. Focusing on the technically demanding process of transitioning between patch recordings and cell extraction, our protocol describes and troubleshoots steps for successfully collecting small interneurons, allowing for multi-modal Patch-seq interrogation of this crucial cell type.

## Highlights

• Detailed step-by-step protocol for collecting cell samples for Patch-seq analysis
• Focus on special challenges posed by studying small interneurons
• Description of specialized “nuclear cork” technique for sample collection
• Extensive troubleshooting guidance

## Graphical Abstract

**Figure:**
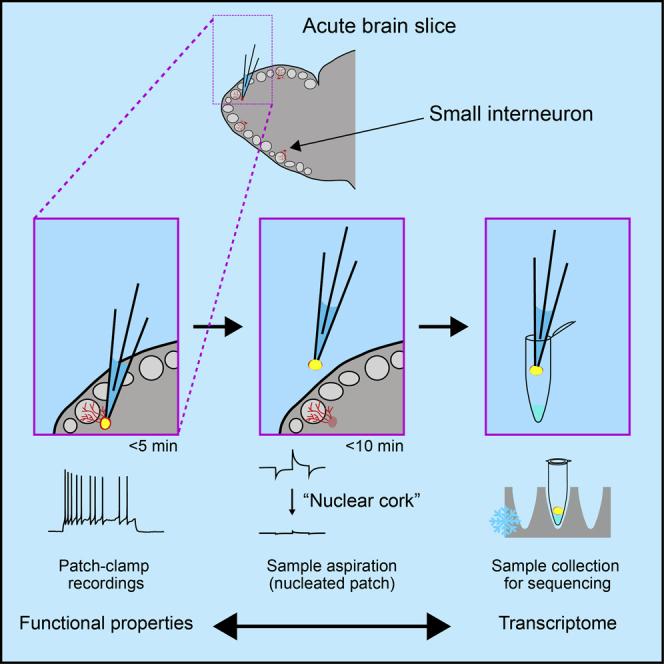

## Before You Begin

Here, we describe a step-by-step protocol to collect samples for Patch-seq. Our experimental workflow is specifically tailored for the recording, collection, and sequencing of small interneurons. In this respect, our approach presents a number of important methodological differences with previously published Patch-seq protocols (Cadwell et al., 2016, 2017; Fuzik et al., 2016; van den Hurk et al., 2018). We obtained Patch-seq data from dopaminergic neurons in the glomerular layer of the mouse olfactory bulb, which we genetically labeled by crossing dopamine transporter (DAT)^IRES*cre*^ mice with a floxed tandem-dimer tomato (tdTomato) reporter line (Byrne et al., 2020; Galliano et al., 2018). Like many other interneurons in the CNS, these cells are usually very small (soma area ~50 μm^2^; Galliano et al., 2018). This small soma size necessitates correspondingly small pipette tip sizes for patch recordings, making it very difficult to subsequently collect intracellular material for sequencing without compromising the high-resistance seal between the neuron and the pipette, a crucial factor in optimizing single-cell sample purity. Our solution to this problem was to develop a nuclear “corking” approach in which, after recording intrinsic neuronal properties, the nucleus is deliberately drawn into the pipette tip and maintained there under a high-resistance seal during sample collection. Details of this novel approach for post-recording extraction of cellular material are outlined here in our step-by-step protocol.

The very first step in this protocol should be to assemble your team. This is ideally a two-person job, preferably involving one specialist electrophysiologist and one specialist molecular biologist. Attempting this protocol alone might just be possible, but will require deep reserves of expertise, focus, patience, dexterity, and perseverance. Also, outline your data analysis strategy in consultation with a statistician or bioinformatician.

Before the day of recording and sample collection, prepare and aliquot all stock solutions, and autoclave, clean, and irradiate all appropriate materials. It is crucial for successful cell collection to ensure that all reagents and materials are free from any contaminants, especially RNases. Purchase all reagents certified nuclease-free (molecular biology grade) and, whenever possible, in solution form. As a general rule, prepare all stocks in single-use aliquots and store all clean material in single-use sealed packs or bundles. Have separate designated areas in freezer, fridge, and cupboards for the exclusive use of Patch-seq materials and solutions and avoid open storage shelves.

Maintain strict sterile and nuclease-free conditions throughout. Change gloves regularly and clean all surfaces with RNaseZap. Step-related tips are provided below.

### Glass Material

**Timing: 2 days**

1. Autoclave twice all glass material to be used for preparing solutions.
2. Dry the autoclaved material by baking at 200°C.

### Prepare DEPC H_2_O and 70% EtOH in DEPC H_2_O for Surface and Tubing Cleaning

**Timing: 2 days**

3. Prepare 0.1% DEPC treated H_2_O following standard protocols.
4. Prepare a 70% EtOH solution using molecular biology grade EtOH and DEPC H_2_O.

### Prepare 1 M KOH Solution Stocks

**Timing: 30 min**

5. Dilute the stock solution (see Key Resources Table) to 1,000 mL in RNase-free H_2_O.
6. Prepare 50 mL aliquots and store at 4°C.

### Prepare Stocks of Sucrose Solution

**Timing: 30 min**

***Note:*** this solution does not need to be 100% RNase-free, as it comes into contact with tissue being sectioned and will therefore contain abundant dead cell debris. However, reasonable clean laboratory practices must be upheld.

7. Prepare 12.5× sucrose solution stock (not including sucrose, glucose, and NaHCO_3_).
  a. Add the dry weights of KCl, MgSO_4_, CaCl_2_, and NaH_2_PO_4_ to ddH_2_O containing magnetic stirrer bar. Stir until fully dissolved. See Materials and Equipment for the detailed recipe.
  b. Store at 4°C until day of use.
8. Prepare 500 mL of the 1× Sucrose solution (Step-By-Step Method Details, step 8a–c). Pour into ice-cube tray and freeze at −20°C. These ice cubes are added to the slicing chamber during acute slice cutting to maintain a low solution temperature at this crucial tissue preparation stage (Step-By-Step Method Details, step 14a).

### Prepare 10× aCSF Stocks and Component Aliquots

**Timing: 1 h**

9. Prepare 10× aCSF stocks (not including glucose, MgSO_4_, and CaCl_2_).
  a. Mix the NaCl, KCl, NaH_2_PO_4_, and NaHCO_3_ stock solutions in nuclease-free H_2_O to the specified volume. See Materials and Equipment for the detailed recipe.
  b. Make 50 mL aliquots and store at 4°C.
10. Make 13.5 mL aliquots of 20% glucose and store at 4°C.
11. Make 1 mL aliquots of MgSO_4_ and store at 4°C.
12. Make 1 mL aliquots of CaCl_2_ and store at 4°C.

### Prepare 1× Intracellular Solution Stocks

**Timing: 1 h**

**CRITICAL:** The intracellular solution (ICS) will be in contact with the internal contents of the cell for the duration of the patch recording and will be collected with the sample. Any contaminants in this solution could potentially be sequenced and the presence of even traces of RNases will negatively impact the quality of the sequencing data generated. Use all certified nuclease-free reagents (molecular biology grade), purchased in solution form when possible, to avoid potential contamination during powder weighing (see Key Resources Table).

***Note:*** Prepare and aliquot this solution in a separate area or room, only used for pre-amplification steps. Work under a PCR cabinet or hood.

***Note:*** Prepare the solution in a glass volumetric flask that was previously rinsed with molecular biology grade nuclease-free H_2_O, wrapped in aluminum foil, autoclaved twice and dried by baking at 200°C.

***Note:*** For preparing the ICS use non-DEPC treated molecular biology grade nuclease-free H_2_O (e.g., Life Technologies cat. no. AM9937; see Key Resources Table).

13. Prepare 1 M sucrose solution using RNase-free H_2_O.
14. Mix the components for 1× ICS. See Materials and Equipment for the detailed recipe.
  a. Weigh the K-gluconate powder and resuspend in molecular biology grade nuclease-free H_2_O in the volumetric flask.

***Note:*** Before weighing, thoroughly clean the balance and surrounding area with RNaseZap and 70% EtOH in DEPC H_2_O. For weighing use a clean twice-autoclaved spatula and an aluminum foil weighing boat.

  b. Add the required volumes of KCl, KOH, MgCl_2_, NaCl, HEPES, EGTA, Sucrose, Na_2_ATP, Na_3_GTP, and Glycogen. Prepare intermediate dilutions for the high concentration stocks to minimize pipetting errors (e.g., use a 1:10 dilution of the 5 M NaCl stock solution in order to pipette 80 μL rather than 8 μL). Mix by stirring the flask. If using a magnetic stirrer, make sure it has been previously rinsed in abundant molecular biology grade, nuclease-free H_2_O, and autoclaved twice.
  c. Check the pH by pipetting a few μL onto a pH strip. Adjust to pH 7.4 with KOH or HCl accordingly.
  d. Complete to the total volume with molecular biology grade, nuclease-free H_2_O.
15. Make 250 μL aliquots and store at −20°C until use.
16. Add 2 μL of RNase inhibitor (20 Units/μL) to one 250 μL aliquot and check the osmolarity. It should be ~290 mOsm. If slightly higher, adjust the amount of glucose in the aCSF, aiming for an aCSF osmolarity that is ~10 mOsm higher than that of the ICS (for example, we commonly added 13.5 mL of 20% glucose solution per 500 mL of aCSF). If the ICS osmolarity is not within the range of ~270–310 mOsm, prepare a new batch of ICS (Troubleshooting 1).

### Prepare Stocks of Channel Blockers

**Timing: 30 min**

17. Make up 2,3-dioxo-6-nitro-7-sulfamoyl-benzoquinoxaline (NBQX), DL-2-Amino-5-Phosphonovaleric Acid (APV) and SR-95531 (Gabazine) as 1,000× stocks in nuclease-free H_2_O (see table below in Materials and Equipment for concentrations).
18. Make 50 μL aliquots and store at −20°C until use.

### Prepare Intracellular Solution Loading Tips

**Timing: 2 days**

19. Wrap a new box of gel loading tips (see Key Resources Table) in aluminum foil and autoclave twice.
20. Store in a separate, dedicated box, or cupboard until use.

### Prepare Glass Capillaries for Electrophysiological Recording

**Timing: 2 days**

21. Prepare bundles of 10 capillaries in 15 mL falcon tubes, wrap in aluminum foil, and autoclave twice.
22. Store in a separate, dedicated box or cupboard until use.
23. Prepare big glass Petri dishes (15 cm diameter) for storing pulled glass capillaries. Rinse in DEPC H_2_O, wrap in aluminum foil, and autoclave twice. Place a band of blu tack (plasticine) along the middle to hold the pipettes. Rinse thoroughly with RNaseZap and 70% EtOH in DEPC H_2_O.

### Prepare the Electrophysiology Rig for Patch-Seq

**Timing:1 day**

***Note:*** If Patch-seq experiments are carried out over a prolonged period, it may be advisable to repeat the following steps at periodic intervals.

24. Replace all tubing and tubing connectors.
25. Remove all sources of physical obstructions on the rig. Tie back any loose wires and ensure clear unobstructed access to all appliances for regular cleaning.
26. If using a setup with a removable recording chamber remove the chamber, clean with ethanol and DEPC H_2_O, and irradiate with UV light. Attach a new, clean glass slide to the bottom of the well.
27. For non-removable or difficult-to-remove parts of the rig, such as the microscope, stage and headstage, clean using tissue paper soaked with 70% EtOH in DEPC H_2_O, removing any build-up of organic or inorganic residue that might be present.
28. Clean the microscope objective lenses by carefully applying a 3% acetic acid solution and gently dabbing it off with optical lens tissue. Repeat this step with DEPC H_2_O.

### Miscellaneous Aliquots

**Timing: 1 h**

29. Prepare 1 mL aliquots of nuclease-free H_2_O. These will be used for rinsing the ICS filtering syringe and gel loading tips.
30. Prepare 200 μL aliquots of RLT plus lysis buffer.

**CRITICAL:** RLT plus buffer contains guanidine thiocyanate, which is harmful. Handle with care. Always use gloves.

## Key Resources Table

**Table:** 

REAGENT or RESOURCE	SOURCE	IDENTIFIER
**Chemicals, Peptides, and Recombinant Proteins**		

DEPC (Diethyl pyrocarbonate)	Sigma-Aldrich	D5758
Acetic Acid (glacial, >99%)	Sigma-Aldrich	695092
NaCl (Sodium Chloride solution 5 M)	Sigma-Aldrich	S5150
KCl (Potassium Chloride solution 1 M)	Sigma-Aldrich	60142
KCl (salt)	Sigma-Aldrich	P3911
MgSO_4_⋅7H_2_O (salt)	Sigma-Aldrich	230391
CaCl_2_ (salt)	Sigma-Aldrich	C4901
NaH_2_PO_4_ (salt)	Sigma-Aldrich	S0751
NaHCO_3_ (salt)	Sigma-Aldrich	S5761
Glucose (solid)	Sigma-Aldrich	G7528
Sucrose (solid)	Sigma-Aldrich	S9378
NaH_2_PO_4_ (Sodium phosphate monobasic solution 5 M)	Sigma-Aldrich	74092
NaHCO_3_ (Sodium bicarbonate solution 7.5%)	Sigma-Aldrich	S8761
H_2_O (UltraPure™ DNase/RNase-free Distilled Water), referred to as RNase-free H_2_O	Invitrogen	10977035
Glucose (Glucose solution 20%)	Sigma-Aldrich	49163
MgSO_4_ (Magnesium sulfate solution 1 M)	Sigma-Aldrich	M3409
CaCl_2_ (Calcium chloride solution 1 M)	Sigma-Aldrich	21115
NBQX	Sigma-Aldrich	N183
APV	Sigma-Aldrich	A5282
SR-95531	Sigma-Aldrich	S106
KOH (for 1 L of 1.0 N solution)	Sigma-Aldrich	1099180001
HCl (1.0 N solution)	Sigma-Aldrich	H9892
MgCl_2_ (Magnesium chloride solution 1 M )	Sigma-Aldrich	M1028
K-gluconate (HPLC grade)	Sigma-Aldrich	G4500
HEPES buffer solution (1 M)	Sigma-Aldrich	83264
Sucrose (HPLC molecular biology grade)	Sigma-Aldrich	84097
Na_2_ATP (Adenosine 5-triphosphate disodium salt solution 100 mM)	Sigma-Aldrich	A6559
Na_3_GTP (Guanosine 5′-triphosphate sodium salt solution 100 mM)	Sigma-Aldrich	G3776
Glycogen (RNA grade 20 mg/mL)	Thermo Scientific	R0551
EGTA (molecular biology grade 0.5 M, pH 8.0)	Alfa Aesar	15405795
H_2_O (Non-DEPC treated, Nuclease-free, molecular biology grade), referred to as “molecular biology grade, nuclease-free H_2_O”	Life Technologies	AM9937
SUPERaseIn RNase inhibitor (20 U/μL)	Life Technologies	AM2694
RLT Plus lysis buffer	Qiagen	1053393

**Experimental Models: Organisms/Strains**		

B6.SJL-*Slc6a3*^*tm1.1(cre) Bkmn*^/J	Jackson Laboratories	Jax stock 006660
B6.Cg-*Gt(ROSA)26Sor*^*tm9(CAG-tdTomato)Hze*^/J	Jackson Laboratories	Jax stock 007909

**Other**		

RNaseZap™ RNase Decontamination Solution	Invitrogen	AM9780
RNaseZap™ RNase Decontamination Wipes	Invitrogen	AM9786
DNA Away	Thermo Scientific	7010
Metal plate for 200 μL PCR tubes	Agilent	410094
Gel loading tips (Microloader™)	Eppendorf	5242956003
P10 filter tips	ClearLine	S1120-3810
UltraCruz® Syringe Filters (for filtering the ICS, 0.22 μm, nuclease-free)	Santa Cruz Biotechnologies	sc-516079
Glass capillaries	World Precision Instruments	1B150F-4
Filters (for patching and mouth pipetting assembly, 0.45 μm)	Biofil	S13PES045B
Aspirator Tube Assembly	Alpha Laboratories	2-000-000

## Materials and Equipment

**Table:** Sucrose Solution

	Volume (mL)	Mass (g)	Final concentration (12.5× stock)	Final concentration (1× working solution)
**12.5× stock solution (1 L)**				

ddH_2_O	1,000 mL	n/a	n/a	n/a
KCl	n/a	4.66 g	62.51 mM	5 mM
MgSO_4_⋅7H_2_O	n/a	6.163 g	25.00 mM	2 M
CaCl_2_	n/a	1.80 g	16.22 mM	1.3 mM
NaH_2_PO_4_	n/a	1.875 g	15.63 mM	1.25 mM

**1× working solution (250 mL)**				

ddH_2_O	230 mL	n/a	n/a	n/a
12.5× stock solution	20 mL	n/a	n/a	n/a
Sucrose	n/a	20 g	n/a	233.7 mM
NaHCO_3_	n/a	0.504 g	n/a	24 mM
Glucose	n/a	0.45 g	n/a	10 mM

**Table:** aCSF

	Stock Concentration	Volume (for 1,000 mL)	Final Concentration (10× stock)	Final Concentration (1× Working Solution)
NaCl	5 M	248 mL	1,240 mM	124 mM
KCl	1 M	50 mL	50 mM	5 mM
NaH_2_PO_4_	5 M	2.5 mL	12.5 mM	1.25 mM
NaHCO_3_	893 mM	291.15 mL	260 mM	26 mM
RNase-free H_2_O	n/a	385.85 mL	n/a	n/a
Added to 1× working solution on day of recording (see text for volumes, Step-By-Step Method Details, steps 9a–c)				
Glucose	20%	n/a	n/a	5.4%
MgSO_4_	1 M	n/a	n/a	2 mM
CaCl_2_	1 M	n/a	n/a	2 mM

**Table:** Intracellular Solution without RNase Inhibitor

	Stock concentration	Volume (for 10 mL)	Final concentration
KCl	1 M	90 μL	9 mM
KOH	1 M	100 μL	10 mM
MgCl_2_	1 M	35 μL	3.5 mM
NaCl	5 M	8 μL	4 mM
K-gluconate	n/a	0.29 g	124 mM
HEPES	1 M	100 μL	10 mM
Sucrose	1 M	285 μL	28.5 mM
Na_2_ATP	100 mM	400 μL	4 mM
Na_3_GTP	100 mM	40 μL	0.4 mM
Glycogen	20 mg/mL	10 μL	20 μg/mL
EGTA	0.5 M	4 μL	0.2 mM
Molecular biology grade, nuclease-free H_2_O	n/a	8.942 mL	n/a

**Table:** Channel Blockers

	Final Conc. (1,000× Stock)	Final Conc. (1× Solution)
NBQX	10 mM	10 μM
APV	50 mM	50 μM
SR-95531	10 mM	10 μM

### Electrophysiology Setup

Successful whole-cell patch-clamp recordings from acutely sliced brain tissue require a suitably equipped and well-maintained patch-clamp setup or “rig.” For an extensive discussion of what makes up a good rig we refer the reader elsewhere (Molleman, 2003). However, the following is recommended as a minimum:

• An upright microscope containing its own or external light source(s) and appropriate optical filters.
• Sufficient magnification to see individual cell bodies in detail; at least a 40× or 63× water immersion objective is recommended.
• An electronic amplifier and digitizer for recording electrical signals from neurons. This should be connected to a means of reading and storing the recorded information such as a computer and/or oscilloscope.
• An air table and air supply to prevent any vibration interference and improve mechanical stability during recording.
• A perfusion system, e.g., peristaltic pump and associated tubing, to circulate external solution through the recording chamber throughout the experiment.
• A reliable local supply of carbogen to oxygenate solutions.
• A means of heating the perfusion solutions to physiologically-relevant temperature, e.g., a temperature-controlled recording chamber and/or in-line solution heater.
• A camera and associated imaging software, for real time visualization of the experiment as well as image capture for later analysis.

***Note:*** Adequate visualization of the cell and patch pipette will become essential during cell extraction steps.

• A stainless-steel or platinum “harp” to hold the slice in place.

### Cell Collection Assembly

**Figure 1:**
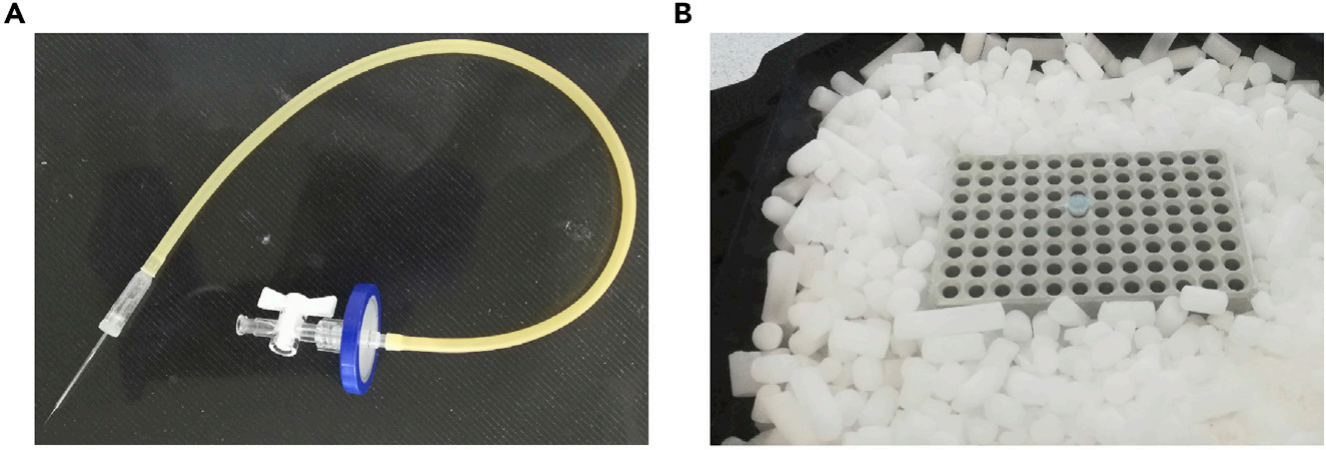
Equipment for Cell Collection (A) Mouth pipetting assembly used for expelling the collected cell into the collection tube. (B) Metal plate sitting on dry ice used for flash-freezing samples.

To efficiently expel the contents of the patch pipette into the collection tube, attach a mouth pipetting assembly to the top of the glass capillary. Cells deposited in 0.2 mL PCR tubes are flash frozen on a metal plate sitting on dry ice (Figure 1).

***Note:*** Using mouth pipetting devices for patching, for cell collection, and for expelling the cell contents into the collection tube allows for finer control of negative and positive pressure than the use of manual syringes. To minimize the potential for contamination we used long tubing and filters (see Figure 1A). In addition, the tubing and the adaptor where the glass capillary is inserted were cleaned daily, with RNA-zap and DNAaway, rinsed in RNase-free 70% EtOH and irradiated under UV light for a minimum of 30 min.

## Step-By-Step Method Details

On the day of recording/sample collection you should have all solution stocks and aliquots already prepared and all materials cleaned and stored in single-use bundles.

Use clean gloves during all steps of all procedures. Change gloves often, especially after cleaning surfaces and/or components and, crucially, before loading ICS into the recording pipette and before retrieving the pipette for transferring the sample into the collection tube.

Maintaining strict cleaning and de-contamination standards is crucial for the successful collection of high-quality Patch-seq samples.

***Note:*** it is best to split the daily solution preparation and slicing between two people. This coordination facilitates the timely preparation of material, aids in avoiding cross-contamination (e.g., the internal solution is prepared and handled by a person that has not been in contact with mouse tissue on the day) and minimizes the time elapsed from slicing to recording and sample collection.

### Daily Pre-recording Electrophysiology Rig Cleaning and Preparation of Materials

**Timing: 30 min**

1. Circulate 50 mL of 70% EtOH in DEPC H_2_O along the perfusion system.

***Note:*** Over time this can weaken the adhesive that seals the recording chamber to its coverslip base. Be aware of this potential issue, and re-seal the chamber when required.

2. Circulate 50 mL of RNase-free DEPC H_2_O along the perfusion system.
3. Wipe all surfaces with RNaseZap and rinse with 70% EtOH in DEPC H_2_O. Pay special attention to the handles, knobs, buttons, keyboards, and mouse that are regularly touched before and during cell extraction.
4. Wash the electrode holder.
  a. Disconnect the electrode holder and disassemble it.
  b. Re-chlorinate the silver wire in bleach for 20 min. Clean with RNaseZap three times. Rinse with molecular biology grade, nuclease-free H_2_O three times. This last wash is crucial as any trace of bleach or RNaseZap can affect cell viability during recording.
  c. Thoroughly clean all the parts of the electrode holder with RNaseZap three times. Rinse with molecular biology grade, nuclease-free H_2_O three times. Air dry inside the PCR cabinet, under UV light for >30 min (Troubleshooting 2).

**CRITICAL:** keeping the electrode wire and electrode holder exceptionally clean is absolutely necessary for collecting good quality samples.

5. Pull recording pipettes.
  a. Wipe all surfaces of the pipette puller thoroughly with RNaseZap and rinse with 70% EtOH in DEPC H_2_O.
  b. Pull glass capillaries to the desired size and shape specifications and place in a clean box with a lid.

***Note:*** For standard borosilicate glass (OD 1.5 mm, ID 0.84 mm, filamented) we used a 2-step pull on an upright puller (Narishige PC-10, load weight: 250g, heat for each step: step 1, 59.6; step 2, 44.9. pull length, 6 mm), which generated pipettes with ~5–6 MΩ resistance and gave identical results for autoclaved versus non-autoclaved glass. Be aware, however, that these parameters will need careful adjustment for individual experimental requirements. Unless we encountered any serious and/or unforeseen issues with pipette resistance or puller settings, we usually pulled all the pipettes used during the whole recording session before starting.

**CRITICAL:** keep the pulled capillaries in the plate with the lid on. Avoid contact with air as much as possible. Always handle capillaries with new, clean gloves.

6. Replace the filter on the patching mouth suction tube (Troubleshooting 3).
7. Wipe Gilson (or other brand) pipettes that will be used for loading the ICS into the recording pipettes with RNaseZap and rinse with 70% EtOH in DEPC H_2_O.

### Prepare 1× Solutions for the Day

**Timing: 30 min**

Starting from single-use stocks and aliquots.

8. Prepare sucrose slicing solution.
  a. Add the dry weight of sucrose (20 g), glucose (0.45 g) and NaHCO_3_ (0.504 g) to 230 mL of ddH_2_O on a stirring plate and wait until everything dissolves.
  b. Bubble with carbogen for ≥10 min to equilibrate the pH.
  c. Add 20 mL of the 10× sucrose stock solution and place on ice. Continue bubbling.
9. Prepare 1× aCSF with channel blockers.
  a. Dilute a 50 mL 10× aCSF aliquot in RNase-free H_2_O to approximately 450 mL.
  b. Add 1 mL of MgSO_4_ 1 M, 1 mL of CaCl_2_ 1 M and 13.5 mL of 20% glucose.
  c. Complete to 500 mL with RNase-free H_2_O.
  d. Bubble with carbogen for ≥10 min to equilibrate the pH.
  e. Add 50 μL each of NBQX, APV, and SR-95531 to 50 mL of aCSF to be used for perfusion during recordings. Continue bubbling.

***Note:*** Antagonists for the main ionotropic glutamate and GABA receptors are included in the aCSF to ensure that the electrophysiological properties recorded from patched cells are truly intrinsic to those neurons, and are not influenced by tonic network activity. If synaptic factors are of interest in particular experiments, some or all of these blockers should be omitted as appropriate.

10. Prepare ICS with RNase inhibitor.

***Note:*** These steps must be performed in a separate pre-amplification area, in a PCR cabinet or hood.

  a. Add 2 μL of RNase inhibitor (20 Units/μL) to a 250 μL aliquot of 1× ICS (final concentration of RNase inhibitor is 0.16 Units/μL).
  b. Rinse a new, sterile 1 mL syringe with molecular biology grade, nuclease-free H_2_O three times.
  c. Using the syringe, filter the ICS through a 0.22 μm filter into a new microcentrifuge tube.
  d. Keep the ICS on ice.

### Prepare the Sample Collection Tubes for Flash-Freezing

**Timing: 45 min**

11. Prepare freezing plate. Clean the metal plate three times with RNaseZap. Rinse with 70% EtOH in DEPC H_2_O. Air dry inside the PCR cabinet, under UV light for >30 min.
12. Prepare the collection tubes. Pipette 2 μL of RLT plus lysis buffer into PCR tubes. Store in a clean box with a lid, at 22°C–24°C, until sample collection.

**CRITICAL:** The collection tubes MUST be compatible with downstream sample processing, as all cDNA synthesis and amplification steps should be carried out in the same tubes, without transferring the sample.

***Note:*** These steps must be performed in a separate pre-amplification area, in a PCR cabinet or hood.

***Note:*** The RLT plus lysis buffer precipitates readily, so these aliquots need to be prepared on the day of use.

***Note:*** the use of a strong lysis buffer is a requirement for accessing the mRNA content inside the nucleus. Its use in this protocol is made possible by the incorporation of mRNA capture steps into the sample processing pipeline (step 23).

13. Wipe an ice bucket with RNaseZap three times and rinse with 70% EtOH in DEPC H_2_O. Load with dry ice and place the clean, dry metal plate on top. Place the lid on.

### Patch-Clamp Recordings

**Timing: 2 h**

The following is a brief description of the process of obtaining whole-cell recordings, focusing on the need to minimize sources of contamination during Patch-seq. More detailed accounts of the technical aspects of whole-cell recordings are available elsewhere (Molleman, 2003; Ogden, 1994; Segev et al., 2016).

The recording protocol needs to be previously optimized to ensure the required functional parameters are obtained in the shortest period of time, to minimize mRNA degradation. As a general rule, do not spend more than 10 min on this step after membrane rupture.

14. Prepare acute slices for electrophysiological recordings.
  a. With appropriate ethical approval, obtain acute slices of the CNS region under study. We use a standard acute slicing protocol (Galliano et al., 2018). For the purposes of Patch-seq the only specific modification is the preparation of clean aCSF (see Materials and Equipment). Dissection and slicing occur in ice-cold oxygenated sucrose solution. Slices are then kept in clean, oxygenated aCSF at 32°C–34°C for 30 min, before being maintained in oxygenated aCSF at 22°C–24°C for the remainder of the experiment. Individual protocols should be optimized for the requirements of particular animal models, tissues, equipment, or experiments.
  b. Perfuse the aCSF extracellular recording solution (containing the channel blockers) through the system and wait for the recording chamber to equilibrate to the correct temperature.
  c. Transfer a brain slice from your recovery chamber to the recording chamber and fix in place with a harp.
15. Load 2 μL of ICS (with RNase inhibitor) into a pulled glass capillary using a gel loading tip.

***Note:*** If the gel loading tip does not have a filter place a P10 filter tip between the pipette and the gel loading tip. We found that the Eppendorf gel loading tips (Microloader™, cat. no. 5242956003) and the ClearLine P10 filter tips (cat. no. S1120-3810) are perfectly compatible.

***Note:*** gel loading tips affect the accuracy of volume measurements. If you need to pipette exactly 2 μL, because the samples will be processed by an automated pipetting station, you can accurately measure the 2 μL using a standard P10 filter tip, place the drop on the back of a microcentrifuge tube lid and aspirate it with the gel loading tip.

16. Obtaining a whole-cell recording.

***Note:*** Obtaining high-quality whole-cell somatic recordings from small neurons usually requires the use of small-tipped, relatively high-resistance pipettes. Typically, we record from olfactory bulb dopaminergic neurons using pipettes with resistance of ~4 MΩ. For Patch-seq recordings, we had to adjust this to ~5–6 MΩ (see Cell extraction). This made obtaining good quality recordings a bit more cumbersome due to higher access resistance, but ensured that the pipette tip was just the right size for cell collection.

**Figure 2:**
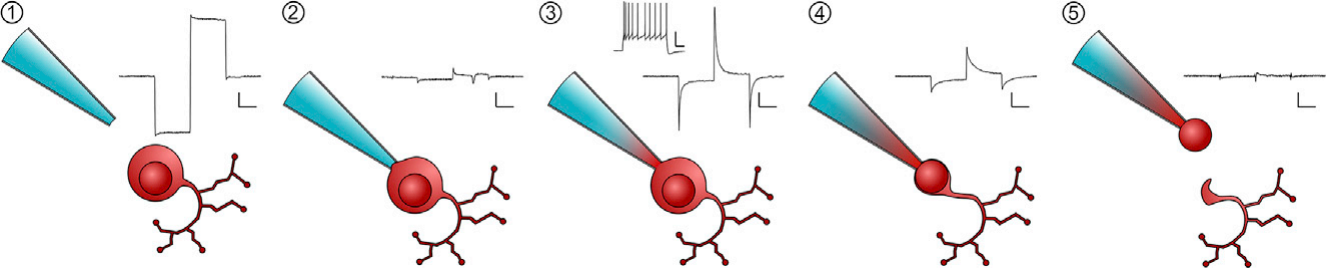
Schematic Timeline for Patch Recordings and Cell Extraction Corresponding representative seal test protocols are shown for each step. Step 1: A clean, unobstructed pipette is lowered into the vicinity of the target cell. Step 2: A giga-ohm seal is formed between the pipette and cell. Step 3: The cell membrane is ruptured yielding electrical access to the inside of the cell. At this point the membrane properties of the cell can be interrogated (e.g., inset trace, showing cell response to 500 ms of depolarizing current injected in current-clamp mode). Step 4: Negative pressure is applied, drawing the cell contents into the pipette and forming a strong seal onto the cell nucleus. Step 5: The pipette is slowly retracted from the tissue bringing the cell nucleus with it. Seal test recordings shown here were obtained by holding the cell at −60 mV and stepping the membrane voltage sequentially to −50 mV and then −70 mV for 10 ms before returning to the holding potential. Scale bar, 10 ms and 1 nA for #1; 10 ms and 200 pA for #2–5; 100 ms and 25 mV for inset in #3.
  f. Evaluate recording parameters online (e.g., leak current, resting potential, etc.). If recordings meet quality thresholds, proceed with sample collection.

***Note:*** Criteria will vary according to individual experimental demands, but should be kept consistent across a study; with the small cells and small pipettes in play here, ours were access resistance ≤33 MΩ with no change >25% across a recording, and holding current ≤100 pA.

  a. Keeping positive pressure on the pipette, lower the electrode into proximity with your chosen cell (Figure 2, step 1).
  b. Zero the voltage offset on the amplifier.
  c. Move the pipette into contact with your cell and remove the positive pressure.

***Note:*** Traditionally this involves lowering the pipette vertically or obliquely toward the surface of the cell until a dimple in the cell membrane is observed. With very small cells the positive pressure from the pipette tip may be enough to significantly distort or even blow the soma away upon approach. Carefully adjusting the level of positive pressure, approaching the cell from the side or at an angle, and/or swiftly switching off the positive pressure when close to the cell soma can help successful seal formation.

  d. Once a giga-Ohm seal is formed, use negative pressure to break into the cell and establish a whole-cell patch configuration (Figure 2, step 2).
  e. Perform the desired electrophysiological experiments (Figure 2, step 3).

### Cell Extraction

**Timing: 10 min**

To maximize single-cell sample purity this procedure must be carried out entirely under good electrical seal (usually >1 GΩ). Ideally, it should not take longer than 10 min, to minimize mRNA degradation.

***Note:*** The specific technique described here, including the “corking” approach, requires some optimization with regards to pipette size, negative pressure applied, and movement of the pipette away from the tissue. Although it might take a little practice, following the steps below is crucial for ensuring successful, clean collection of the recorded interneuron’s intracellular contents. In particular, collection of the cell nucleus significantly increases the cDNA yield (Cadwell et al., 2016; Troubleshooting 4, 5, 6, 7, and 8). Ensuring a good “cork” of the nucleus in the pipette tip allows the nucleus to be collected with a small-tipped pipette without losing the high-resistance seal which optimizes sample purity.

17. Collect the cell content, including the nucleus, using the recording pipette (Figure 2, steps 4 and 5).
  a. Apply negative pressure until the cell has reduced significantly in size. This may take up to several minutes, and with small-tipped pipettes it may require constant suction far stronger than that required to initially obtain a gigaseal. If electrical seal begins to fail, release pressure and allow the cell to recover.
  b. When most of the cell contents have been suctioned into the pipette, begin to manipulate the pipette into proximity with the nucleus. Identifying the nucleus’ location is helped by its generally darker appearance and its more solid presence; it does not move or distort as readily as the plasma membrane in response to pipette manipulations. Continue to apply negative pressure and monitor electrical resistance to observe a change in access resistance when the seal is formed on the nucleus.
  c. Slowly begin to move the pipette away from the tissue. Raise the pipette vertically from the slice in stages, allowing time at each step for the electrical seal to stabilize. If the previous steps were successful the nucleus should be seen accompanying the pipette tip (“corking”), if this is not the case, lower the pipette again in line with the cell body and repeat steps a-b (Troubleshooting 4).

**CRITICAL:** Due to the small size of olfactory bulb dopaminergic neurons, in order to obtain a significant amount of mRNA for proceeding with cDNA synthesis and sequencing library preparations, we needed to ensure that we collected the cell nucleus.

***Note:*** we optimized this step for consistently obtaining a nucleus “cork” during cell extraction. We achieved this by using smaller tip pipettes (~5–6 MΩ) for recording so the nucleus was slightly bigger than the tip and so would generally not be sucked into the pipette.

  d. Finally, as the pipette is extracted from the tissue along with the cell body/nucleus, it may still be connected to the tissue via intact but stretched neuronal processes. Significant care is required here to prevent rupture of the cell and loss of electrical seal due to the tearing of these processes. Move the pipette slowly away from the tissue until the nucleus/soma can be confidently seen to be separated from the slice. Only after this is confirmed is it safe to remove the pipette from the solution. (Figure 2; Troubleshooting 4, 5, 6, 7, and 8).
  e. Check that there is no debris attached to the outside of the pipette (Troubleshooting 9).
18. Transfer the cell to the collection tube.
  a. Remove the glass capillary, or detach the entire pipette holder assembly from the headstage (this will depend on the holder specifications).

***Note:*** Removing the glass capillary from the pipette holder does not affect the small volume of liquid at the tip. It is safe to remove it from the holder.

  b. Quickly bring the tip of the recording pipette to the bottom of a PCR collection tube, containing 2 μL of lysis buffer.

**CRITICAL:** It is essential that the tip of the capillary does not touch anything before contacting the lysis buffer. Special attention must be paid to avoid touching the walls of the collection tube (Troubleshooting 10).

  c. Gently break the tip of the capillary against the bottom of the collection tube.
  d. Apply positive pressure to expel the whole of the pipette content into the lysis buffer. Avoid making bubbles.

***Note:*** If in the previous step the glass capillary was removed from the pipette holder, a mouth pipetting assembly can be quickly placed on the top of the glass capillary to apply positive pressure (Figure 1A).

19. Quickly place the tube on the metal plate sitting on dry ice to flash-freeze the sample (Figure 1B).
20. Assign a sample collection score to the cell, based on successful collection of the nucleus, extraction time, quality of seal, presence of debris on the outside of the pipette and successful depositing into lysis buffer (Figure 3; Troubleshooting 4, 5, 6, 7, 8, 9, and 10).

**Figure 3:**
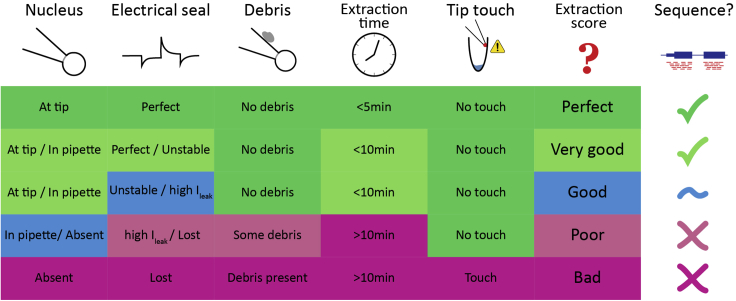
Scoring System for Assessing Cell Extraction Perfectly extracted cells must meet all the criteria outlined: successfully extracted nucleus, seal maintained throughout the whole extraction process, no debris observed on the outside of the pipette, no contact of the tip of the pipette with any surface before it reaches the lysis buffer in the collection tube, and less than 10 min duration for the entire extraction process. Assign a score to each cell and only process for sequencing cells with “perfect” or “very good” scores. Cells with “good” scores can also be sequenced if, for example, sample numbers are low or there is particularly interesting physiology.
21. Store samples at −80°C until processing.
22. Collect negative controls during each recording/collection session.
  a. Load a pipette with ICS and lower to the tissue as if aiming to perform a recording.
  b. After making contact with the tissue, retract the pipette and transfer the contents to a collection tube.
  c. Flash-freeze the sample and store at −80°C until further processing.

**Pause Point:** Frozen samples can be stored at −80°C for up to 6 months before further processing.

### mRNA Capture and scRNA-Seq

**Timing: 1–2 weeks**

The focus of this protocol is on Patch-seq sample collection: the steps required to obtain high-quality material for sequencing after whole-cell recordings in small interneurons. We do not aim to provide a comprehensive description of the downstream procedures needed to generate and analyse scRNA-seq or other -omic data. Detailed protocols for this are published elsewhere (Macaulay et al., 2016; Picelli et al., 2014; Troubleshooting 11, 12, and –13). Instead, here we briefly outline a possible workflow based on our own experiments.

23. Follow the G&T protocol to capture poly-adenylated mRNA (Macaulay et al., 2016).

***Note:*** Although this adds a significant number of steps to sample processing, it presents a number of advantages. First, this step is necessary for removing any traces of the RLT plus lysis buffer. Second, it helps in controlling the total volumes of subsequent reactions. Third, it generates a separate gDNA fraction that is available for pursuing questions at the genomic level.

***Note:*** before starting the G&T procedure, add 1 μL of spike-in RNA (typically, 1:1–2 million dilution, but this will depend on the cell type) to each sample. These artificial RNAs will serve to control for technical artifacts during cDNA synthesis, library preparation and sequencing (Jiang et al., 2011).

24. Perform cDNA synthesis and amplification following the Smart-Seq2 protocol (Picelli et al., 2014).
25. Quantify and quality control the cDNA using standard procedures (e.g., Qubit and bioanalyzer) and by running qPCRs for relevant marker genes.

***Note:*** extensive cDNA quality control (Qubit + bioanalyzer + qPCRs; Figure 5) is highly recommended during the initial troubleshooting stages, when Patch-seq workflows are being established anew. This will provide the necessary feedback for outlining the cell collection scoring criteria.

26. Prepare sequencing libraries by tagmentation using the Illumina Nextera XT kit and introduce sample indexes.
27. Quantify and quality control the pooled libraries using standard procedures (e.g., Qubit, bioanalyzer, and qPCR quantification).
28. Sequence the libraries on the appropriate Illumina platform (e.g., HiSeq2500). Typically, samples are pooled to obtain ~2 million reads per cell. For cell types with complex transcriptomes (e.g., neurons) this sequencing depth will ensure quantitative gene expression data for >5,000 genes, comprising highly, averagely and lowly expressed genes, and ensuring these include genes coding for ion channels and transporters which are of particular interest for experimental questions addressed using Patch-seq protocols.
29. Demultiplex, QC, map and quantify the sequencing reads following standard protocols (Vieth et al., 2019).
30. Perform sample-level QC to identify successfully sequenced samples and eliminate low-quality cells (see examples in Figure 5). Continue with your data analysis pipeline (Luecken and Theis, 2019; Lun et al., 2016).

## Expected Outcomes

**Figure 4:**
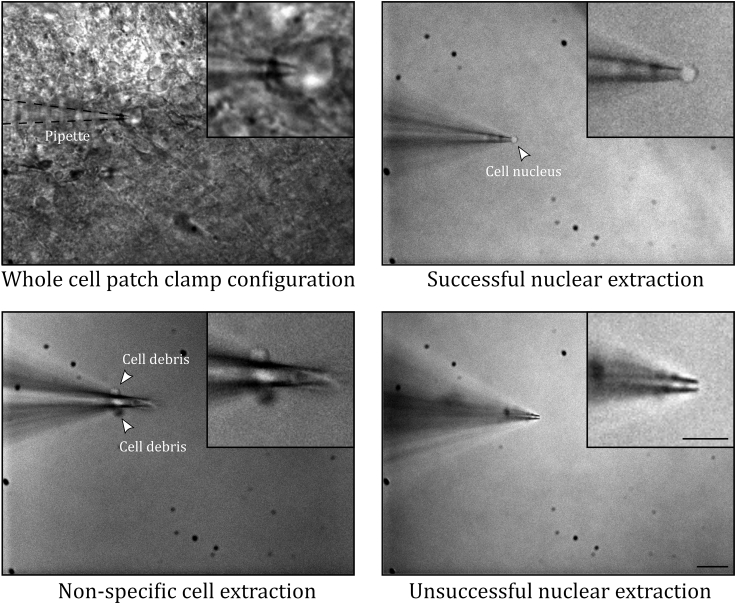
Example Images of Patching and Cell Collection Top left: Whole-cell patch-clamp recording of a glomerular layer interneuron from an acute slice of juvenile mouse olfactory bulb. Top right: Pipette held above the slice after a successful extraction of the cell nucleus. Bottom left: Post-recording pipette with non-specific cellular debris attached to the side of the glass. Bottom right: Post-recording pipette where the nucleus has become dislodged from the pipette tip during the extraction procedure. Scale bar, 20 μm for the main panels and 10 μm for the insets.

Adapting patch recordings of very small interneurons for compatibility with sample collection for Patch-seq presents a number of challenges that impact the recording itself, the sample collection procedure, and the number of successfully collected cells per session. Because we performed recordings with smaller than usual pipettes to improve the cell collection step, the rate of successfully patched cells was lower than usual. This elevated difficulty in obtaining patch recordings, coupled with the need to study specific fluorescently-labeled neurons and the practical difficulties inherent in the cell extraction process for small interneurons, meant that we typically collected around 4 good quality cells per day, among roughly equal numbers of failed attempts (Figure 4).

**Figure 5:**
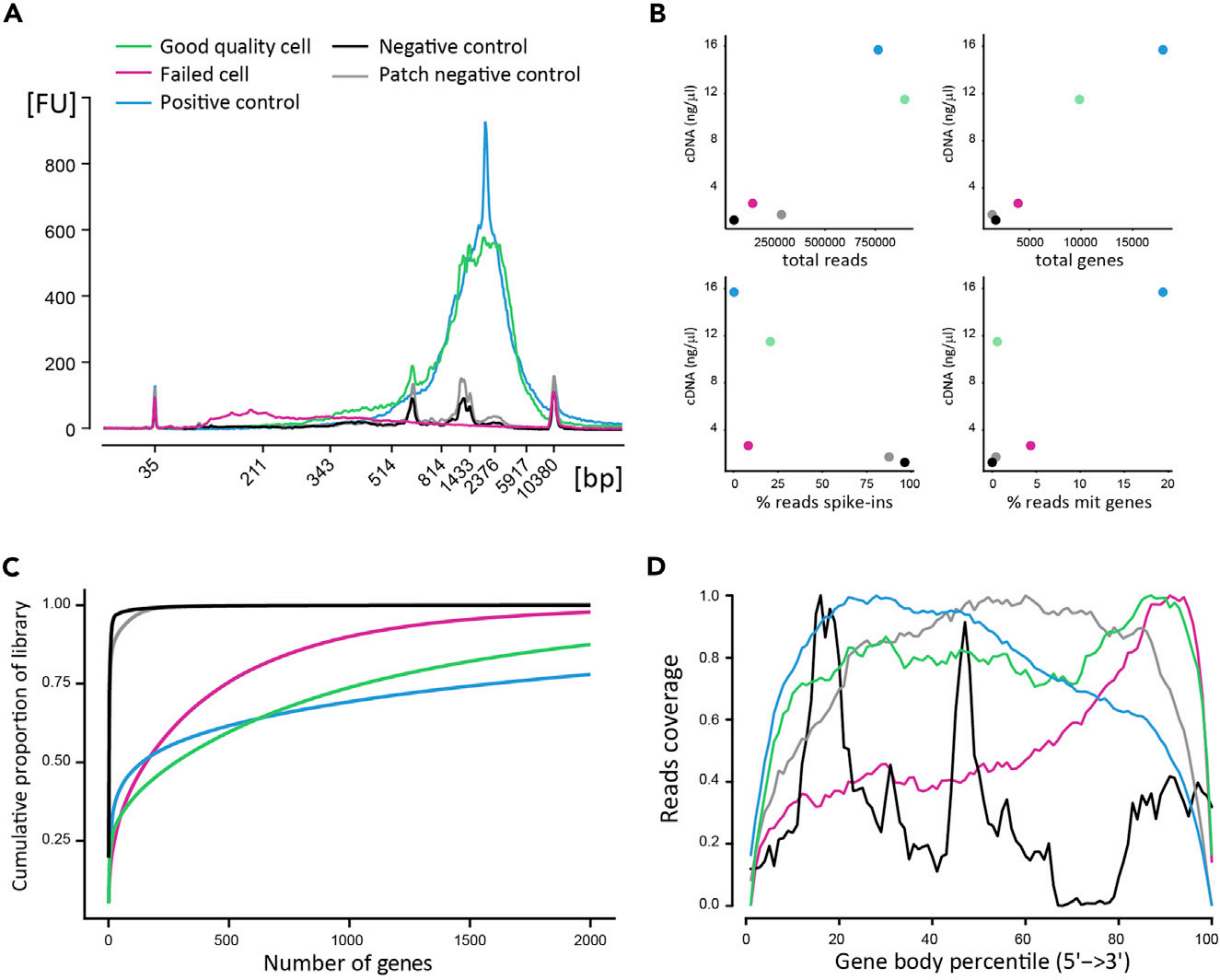
Data from Five Representative Example Patch-Seq-ed Cells, Illustrating Different Potential Outcomes in Quality Control Metrics for cDNA Preparation and scRNA-Seq (A) Bioanalyzer capillary electrophoresis traces. (B) scRNA-seq quality metrics, showing per sample cDNA concentration, total number of mapped reads per cell, total number of genes detected per cell, percentage of reads mapping to technical ERCC spike-ins per cell and percentage of reads mapping to mitochondrial genes per cell. (C) Library complexity depicted as the cumulative proportion of the total number of genes detected per cell. (D) Reads mapping along gene body length showing full-length cDNA coverage for good quality samples. Green, good quality cell; red, low-quality cell; blue, positive control; black, negative control; gray, patch negative control.

Using a number of electrophysiological parameters and our cell extraction scoring system, we could decide, at the time of collection, whether each individual sample was of good enough quality to warrant moving ahead to cDNA synthesis and sequencing (Figure 3). This presents an advantage against other single-cell collection methods (e.g., FACS) for which there is no experimental certainty on the quality of each individual sample prior to cDNA synthesis and sequencing. Overall, collected samples that were subsequently sequenced yielded excellent quality sequencing data, successfully quantifying thousands of genes per cell (>80% of sequenced cells passed all sequencing QC metrics; see “Good quality cell” example in Figure 5).

Our sample collection and processing workflow included a series of steps to pull-down the mRNA, following the G&T protocol. This allowed for flexibility in the volume of ICS used for recording and the lysis buffer used for sample collection. More importantly, the protocol produces a separate fraction, containing the genomic DNA, that is now available to pursue new experimental questions.

Carefully choosing the recording protocol, following the optimized cell collection steps outlined above, and sequencing at enough depth to obtain accurate expression information for relevant genes (e.g., ion channels and membrane transporters) will allow for accurate analysis of the relationships between functional parameters and gene expression patterns.

## Limitations

The collection of samples for Patch-seq is extremely low throughput, especially in comparison with other scRNA-seq approaches. In addition to the Patch-seq-ed experimental samples, we strongly recommend performing scRNA-seq, in parallel, on the same neuronal type/stage using a higher throughput sample collection method (e.g., FACS or manual sorting). This will greatly enhance data analysis strategies and improve statistical power. Also, performing a whole tissue scRNA-seq experiment (e.g., 10X genomics) to analyze gene expression in all constituent cell types of the brain region in question, including those targeted for Patch-seq, will allow for sample specific quality and contamination control (Tripathy et al., 2018).

A number of contamination and quality control measurements make the patching procedures more laborious than a stand-alone electrophysiology protocol, potentially reducing the number of successfully collected samples per session. First, because during electrophysiological recordings the intracellular environment is exposed to the ICS and the recording electrode, these two components are crucial to obtaining good quality samples. Keeping them absolutely free of contaminants, including nucleases, is laborious but a requirement for collecting useful samples. Second, during cell extraction extreme caution must be observed in maintaining an electrical seal. This guarantees that the intracellular environment never comes into contact with the circulating aCSF. Because this external solution is bathing a tissue slice with dying cells and debris from already dead cells, it is a major source of cross-contamination. Sticking to the “extraction under seal” requirement greatly reduces the number of successfully collected cells per session, especially during early training stages. For our sample collection process, because we were patching with smaller than usual pipettes, maintaining the seal was made all the more complex and benefited from this experiments being performed by a highly skillful and experienced electrophysiologist. With practice, this step becomes less of a bottleneck.

Due to the small size of olfactory bulb dopaminergic neurons, in order to obtain a significant amount of mRNA for proceeding with cDNA synthesis and sequencing library preparations we needed to ensure that we collected the cell nucleus. Efficient access to nuclear mRNA requires a stronger lysis buffer, like the RLT plus buffer used here. This presents the limitation of seriously affecting downstream reactions. cDNA synthesis cannot directly be performed on a cell collected on RLT plus buffer, as the denaturation agents present will inactivate the retrotranscriptase. To solve this issue, we introduced extra sample processing steps for capturing and washing the mRNA, following the G&T protocol (Macaulay et al., 2016). The downsides of this are the added cost of reagents and that the lengthier protocol meant that samples could not be processed on the day of collection. We stored all our collected samples at −80°C and processed them in batches. Storage in this way was not associated with any change in sequencing quality up to our longest storage period of almost 7 months (Pearson correlation with days in storage for “good” cells; total reads per cell, R = −0.07, p = 0.57, n = 68; total genes per cell, R = 0.15, p = 0.21, n = 68).

The G&T protocol, though lengthier and more costly, presented a much welcomed advantage. Because the mRNA is pulled down with magnetic beads and the starting volume consequently does not affect downstream reactions, we could perform electrophysiological recordings using a significantly higher volume (2 μL) of ICS than those previously reported for Patch-seq experiments (0.1–0.9 μL; Cadwell et al., 2017; Fuzik et al., 2016; Troubleshooting 14). Finally, in our hands, and in line with previous publications (Macaulay et al., 2016) the incorporation of the G&T steps to our sample processing pipeline did not affect the quality and/or quantity of scRNA-seq data obtained. Overall, the incorporation of G&T steps presented more advantages than limitations. However, these steps for mRNA capture and wash are not an absolute requirement for the preparation of scRNA-seq libraries. If the G&T protocol is not possible or preferred, samples should be collected in a milder lysis buffer (e.g., 0.2% Triton X-100) and the volume of ICS used should be lowered so as to not hamper the cDNA synthesis reaction.

## Troubleshooting

### Problem 1

The osmolarity of the ICS is very high, and stable recordings are impossible (Before You Begin, step 16).

### Potential Solution

The addition of RNase inhibitor and glycogen to the ICS can significantly alter the osmolarity of standard recording solutions. Small excesses in ICS osmolarity (<20 mOsm) can be offset by adding additional glucose to the aCSF. Larger differences can be troubleshot by making “mock” solutions first that do not contain expensive nucleotides, to establish the effect of glycogen and RNase inhibitor on ICS osmolarity.

### Problem 2

A voltage offset is present which is not removed when pipette is “zeroed” (step 16b).

### Potential Solution

Because the pipette holder is disassembled and thoroughly cleaned every day, occasionally a drop of liquid can remain lodged in the smaller nooks and crannies. Ensure that every part of the holder is perfectly dry before re-assembling.

### Problem 3

Loss of pressure or poor pressure control through the pipette holder (step 16c).

### Potential Solution

Ensure the tubing between the pressure system and head stage/pipette holder is clean and unobstructed. Repeated strong pressure can affect the integrity of the filter. For this reason, and as a contamination control measure, change the filter daily.

### Problem 4

Cell extraction fails. The nucleus was clearly left behind when removing the pipette (step 17).

### Potential Solution

During the extraction procedure, move the pipette closer to the nucleus and apply negative pressure until a seal is formed on the nucleus. This is observed as a change in access resistance (Figure 2). Continue applying negative pressure, pulling the nucleus away from the rest of the cell. This will leave all projections behind, but will extract the nucleus under seal (i.e., nucleated patch), which contains most of the cell’s mRNA. When the cell is successfully out of the tissue, it should still be under seal. In DIC, the nucleus is easily spotted when it was successfully pulled out of the tissue and it is “corking” the pipette (Figure 4, top right). If the nucleus was nonetheless left behind, and not clearly visible “corking” or inside the tip of the pipette (Figure 4, bottom right), discard the sample.

### Problem 5

Cell extraction fails. Electrical seal clearly lost during cell extraction (step 17).

### Potential Solution

This problem has no solution for that specific sample. The cell must be discarded. During the next cell collection attempt, apply milder negative pressure and wait for the cell to accommodate. Different interneuron types may require slightly different extraction techniques in terms of pipette tip size, negative pressure strength, and/or speed of pipette retraction. Optimizing the extraction technique in advance of commencing the experiment is highly recommended.

### Problem 6

Cell extraction fails. The cell was clearly left behind when removing the pipette (step 17).

### Potential Solution

This is a major problem with cells with somas that are solidly embedded within local neuropil, as is the case for olfactory bulb dopaminergic neurons. Here, it pays off to take slightly longer to pull the cell out of the tissue. Retract the pipette only slightly, wait for the seal to stabilize and keep an eye on the cell making sure there are no projections that are stretching and still attached to the tissue. With time, those connections will break and seal off and an intact sample, under seal, will be collected. If the problem persists, try modifying the pipette tip size. If the pipette tip is small, sufficient traction of the cell nucleus is difficult; too large and the nucleus will not become lodged in the tip and the seal may be lost.

### Problem 7

Cell extraction fails. No noticeable amount of cytoplasm is observed going into the pipette and there is no detectable change in cell size (step 17).

### Potential Solution

This is a major problem for small, compact neurons such as olfactory bulb dopaminergic neurons. After finishing the recordings, and without moving the pipette, apply slight negative pressure to suck the small amount of cytoplasm into the pipette. We usually observed some red fluorescence inside the pipette at this step, without actually noticing any change in the size of the cell soma. At this point, this is not an extraction failure. These interneurons are so small that their somas are virtually all nucleus, hence the lack of size shrinkage when suctioning the cell content. As described in the step-by-step section, the approach to obtain enough sequencing material is to ensure the collection of the nucleus.

### Problem 8

Seal collapses at a very late stage of extraction (step 17).

### Potential Solution

Even after perfect extraction, a sudden collapse of the seal can occur, often accompanied by a change in the visual appearance of the cell from a smooth ball to a more translucent and defined nucleus shape. If this happens when the pipette is far away from the slice, and is only partial, with significant nucleus corking maintained, the cell might still be successfully included in future analyses. If not, discard the sample.

### Problem 9

Cell collection fails. Tissue debris is observed on the outside surface of the pipette (Figure 4, bottom left) (step 17).

### Potential Solution

After the cell has been successfully extracted from the tissue, under electrical seal, inspect the pipette, by moving the focus up and down, to ensure there is no debris attached. If debris is observed, then slowly move the pipette around to see if it detaches. Alternatively, as a last resort, because it can dislodge the nucleus and/or rupture the seal, move the pipette outside of the solution and re-submerge it. Visually inspect the pipette to confirm debris was removed and ensure that electrical seal was maintained throughout. If either of these fails, discard the sample.

If debris is regularly observed, make sure the aCSF was prepared with high-quality water (see Key Resources Table) and that the tubing is clean. If necessary, prepare new aCSF stocks and change all the tubing for the perfusion system. Also consider targeting cells that are closer to the slice surface.

### Problem 10

Cell collection fails. The tip of the pipette touched another surface (e.g., upper side wall of collection tube; Figure 3) (step 18).

### Potential Solution

Discard the sample. Also, drink less coffee.

### Problem 11

Low (or no) cDNA obtained for an individual sample (steps 24 and 25).

### Potential Solution

Sample collection and/or storage failed. Only process and sequence samples that meet all the sample collection criteria: (e.g., seal maintained throughout the extraction, cell quickly placed into tube and frozen; Figure 3).

### Problem 12

Low (or no) cDNA obtained for an individual sample (steps 24 and 25).

### Potential Solution

Double-check all solution preparation and sample collection steps to ensure all contamination control and nuclease-free requirements are met. Prepare new solution stocks and single-use aliquots. Ensure the pre-amplification area and PCR cabinet are free of contaminants. Re-clean all equipment and benchtop surfaces. Change gloves more frequently, especially before loading the ICS into the recording pipette and during sample collection. Always run positive control samples in parallel.

### Problem 13

Low (or no) cDNA, low (or no) reads/genes obtained for an individual sample (steps 24 and 25).

### Potential Solution

mRNA capture, cDNA synthesis and/or library preparation may have failed (see “Failed cell” example in Figure 5). The preparation of scRNA-seq relies on the capture and amplification of a very small amount of starting material. There are multiple reasons why any of these steps may fail, resulting in sample loss. These troubleshooting aspects are beyond the scope of the present protocol. Readers should refer to relevant publications (Macaulay et al., 2016; Picelli et al., 2014), or contact the authors for in-depth discussions.

### Problem 14

Difficulties patching with lower than usual volume of ICS (step 16).

### Potential Solution

Load the patching pipette with 2 μL of ICS and bring the volume back down for the cDNA synthesis reaction by first performing an mRNA capture step.

## Resource Availability

### Lead Contact

Further information and requests for resources and reagents should be directed to and will be fulfilled by the Lead Contact, Matthew S. Grubb (matthew.grubb@kcl.ac.uk).

### Materials Availability

No new mouse lines or materials were generated in this study.

### Data and Code Availability

Sequencing data corresponding to the example cells depicted in Figure 5 is available at NCBI GEO (GSE151709). Data analysis code is available upon request.
